# Crystal structure of (*RS*)-4-(3-carb­oxy-1-ethyl-6,8-di­fluoro-4-oxo-1,4-di­hydro­quinolin-7-yl)-2-methyl­piperazin-1-ium 3-carb­oxy-5-fluoro­benzoate

**DOI:** 10.1107/S2056989018016961

**Published:** 2019-01-01

**Authors:** Sheng Feng, Gui-Liang Zhu, Jia-Jia Sun, Chen Chen, Zhi-Hui Zhang

**Affiliations:** a School of Environmental and Safety Engineering, Changzhou University, Changzhou, 213164, People’s Republic of China; b Jiangsu Key Laboratory of Advanced Catalytic Materials and Technology, Changzhou, University, Changzhou 213164, People’s Republic of China

**Keywords:** crystal structure, proton transfer, lomefloxacin, hydrogen-bonded network

## Abstract

In the title organic salt, C_17_H_20_F_2_N_3_O_3_^+^·C_8_H_4_FO_4_^−^, proton transfer leads to one protonated lomefloxacin mol­ecule (HLf^+^) and one 3-carb­oxy-5-fluoro­benzoate (5-F-Hip^−^) anion in the asymmetric unit. The HLf^+^ cation is bent, with a dihedral angle of 38.3 (1)° between the quinoline ring and the piperazinium moiety. In the crystal, two kinds of N—H⋯O and O—H⋯O hydrogen-bonded chains cross-link each other to produce a three-dimensional network structure that is additionally stabilized by weak C—H⋯O and C—H⋯F hydrogen bonds, as well as π–π inter­actions. The methyl group attached to the piperazinium ring is disordered over two sets of sites [refined ratio: 0.645 (5):0.335 (5)], indicating the presence of both enanti­omers of the cation in the structure.

## Abstract 2

Reaction of lomefloxacin [(*RS*)-4-(3-carb­oxy-1-ethyl-6,8-di­fluoro-4-oxo-1,4-di­hydro­quinolin-7-yl)-2-methyl­piperazine, Lf] with 5-fluoro­isophthalic acid leads to a charge-assistant hydrogen-bonding network between HLf^+^ cations and 3-carb­oxy-5-fluoro­benzoate anions.

## Chemical context   

Lomefloxacin [Lf; systematic name: (*RS*)-4-(3-carb­oxy-1-ethyl-6,8-di­fluoro-4-oxo-1,4-di­hydro­quinolin-7-yl)-2-methyl­piperazine] belongs to the fluoro­quinolones that represent an important family of highly effective broad-spectrum anti­bacterial agents (Ross & Riley, 1990 ▸; Reddy *et al.*, 2011 ▸; Huang *et al.*, 2013 ▸). Lomefloxacin is very useful for the treatment of a variety of infections, although its therapeutic action as a drug is limited due to poor aqueous solubility (1.03 mg ml^−1^, Ross & Riley, 1990 ▸). Using salts of lomefloxacin may overcome this problem. Several binary and ternary salts of lomefloxacin have been reported with supra­molecular arrangements of the cationic and anionic moieties, such as the terephthalate (Zhou *et al.*, 2006 ▸), isophthalate (Zhang *et al.*, 2015 ▸), picrate (Jasinski *et al.*, 2011 ▸) or hydro­chloride (Holstein *et al.*, 2012 ▸). However, the number of compounds related to solubility improvement is rather limited (Zhang *et al.*, 2015 ▸).
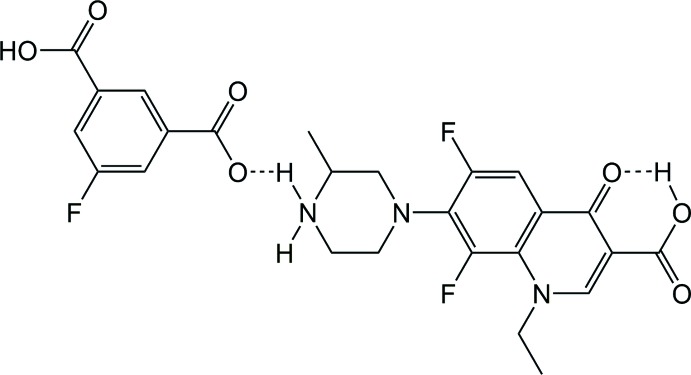


In this context, we have used 3-carb­oxy-5-fluoro­benzoic acid (5-F-H_2_ip) for a proton-transfer reaction, and report here synthesis and crystal structure of the produced salt (HLf)^+^·(5-F-Hip^−^), (I).

## Structural commentary   

**Figure 1:**
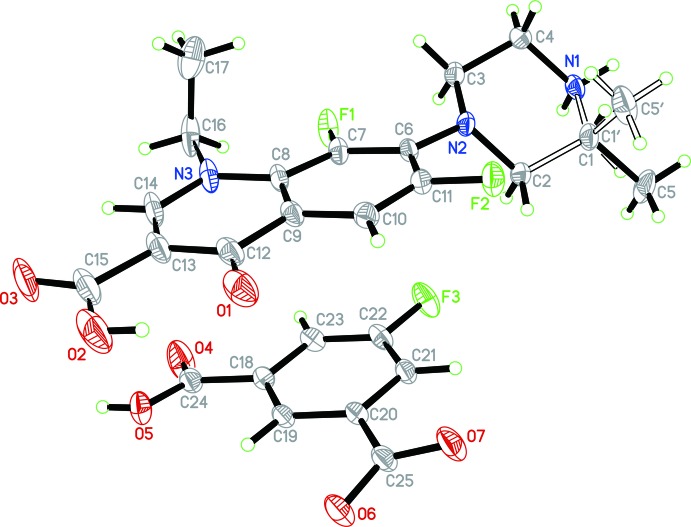
Mol­ecular structures of the cation and anion in the title salt. Displacement ellipsoids are drawn at the 30% probability level.

The structures of the mol­ecular entities of (I) are displayed in Fig. 1 ▸. Unlike other lomefloxacin salts (Zhang *et al.*, 2015 ▸), the title compound reveals no guest solvents residing in the crystal structure. In the asymmetric unit, there is one HLf^+^ cation and one 5-F-Hip^−^ anion, *i.e*. only one proton has been transferred from the free acid. Within the HLf^+^ moiety, a non-planar conformation of the mol­ecule is formed with a dihedral angle of 38.3 (1)° between the aromatic ring plane and the piperazinium ring (the latter exhibits a chair conformation). An intra­molecular *S*(6) hydrogen-bonding pattern (Etter *et al.*, 1990 ▸) is found between the carb­oxy­lic group and the carbonyl O atom (O2—H2⋯O1; Table 1 ▸). The 5-F-Hip^−^ anion is nearly planar (r.m.s. deviation = 0.132 Å), with the highest deviation of 0.2645 (13) Å for the carboxyl­ate O6 atom.

## Supra­molecular features   

**Figure 2:**
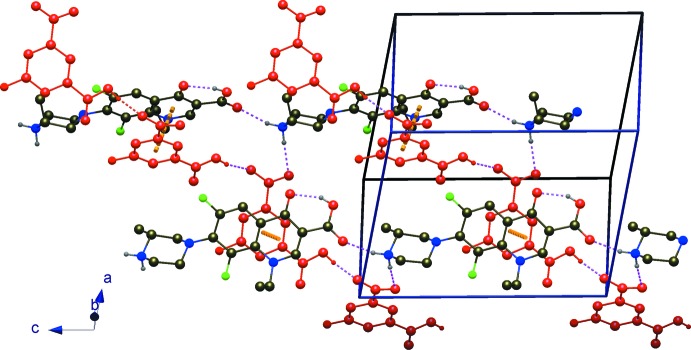
A perspective view of (I) showing the N—H⋯O and O—H⋯O hydrogen-bonding inter­actions (dotted lines) between the two kinds of chains. ‘Acidic’ chains, *i.e.* chains involving only the anion, are shown in red for clarity.

In the crystal structure, N1—H1*A*⋯O3^iii^ inter­actions between the amino function of the piperazinium moiety and the non-protonated O atom of the carb­oxy­lic group of a neighboring HLf^+^ cation result in a head-to-tail chain motif with descriptor *C*(13). Adjacent 5-F-Hip^−^ moieties also form a head-to-tail chain, based on a *C*(8) pattern, involving O5—H5⋯O7^i^ bonds between the carb­oxy­lic acid function and the carboxyl­ate function. The two kinds of chains inter­link with each other through N1—H1*B*⋯O6^ii^ inter­actions between the second H atom of the amino group of the cation and one of the carboxyl­ate O atoms of the anion to form a three-dimensional network structure. Within this array (Fig. 2 ▸), additional weak C—H⋯O and C—H⋯F inter­actions are present (Table 1 ▸) as additional stabilization forces, along with π–π inter­actions between fluoro­quinolone benzene rings of the cations and and phenyl rings of the anions with a centroid-to-centroid separation of 3.7895 (12) Å.

## Database survey   

Two crystal structures (Zhang *et al.*, 2015 ▸) based on lomefloxacin and isophthalic acid have been reported in the CSD (Verson 5.39; Groom *et al.*, 2016 ▸) viz. CURKAD [4-(3-carb­oxy-1-ethyl-6,8-di­fluoro-4-oxo-1,4-di­hydro­quinolin-7-yl)-2-methyl­piperazin-1-ium 3-carb­oxy­benzoate hydrate] and CURKIL [4-(3-carb­oxy-1-ethyl-6,8-di­fluoro-4-oxo-1,4-di­hydro­quinolin-7-yl)-2-methyl­piperazin-1-ium 2,6-dioxo-1,2,3,6-tetra­hydro­pyrimidin-4-olate isophthalic acid methanol solvate monohydrate]. Both CURKAD and the title compound are proton-transfer compounds from isophthalic acids to the piperazine NH groups. In the structure of CURKIL, the isophthalic acid moiety remains protonated, and co-crystallized barbituric acid is the proton donor in this case. With respect to the supra­molecular networks in these structures, the contribution of the extra fluorine atom in (I) leads to additional hydrogen bonds of the type C—H⋯F.

## Synthesis and crystallization   

A methanol solution (6 ml) of 5-fluoro­isophthalic acid (5-F-H_2_ip; 20 mg, 0.1 mmol) was mixed with a slurry of lomefloxacin (Lf) (35 mg, 0.1 mmol) in 5 ml water under stirring. The mixture was exposed to ultrasound for *ca* 20 min, and was then filtered and left to slowly evaporate. Colourless block-like single crystals suitable for X-ray analysis were obtained after several weeks. Yield: 65% (35 mg, based on Lf). Analysis calculated for C_25_H_24_F_3_N_3_O_7_: C, 56.08; H, 4.52; N, 7.85%. Found: C, 56.06; H, 4.50; N, 7.82%. FT–IR (KBr pellet, cm^−1^): 3431*b*, 3070 (*w*), 2475 (*w*), 1718 (*s*), 1620 (*vs*, 1539 (*m*), 1456 (*s*), 1371 (*m*), 1275 (*s*), 1090 (*m*), 959 (*m*), 901 (*w*), 766 (*m*), 689 (*m*), 521 (*w*).

## Refinement   

Crystal data, data collection and structure refinement details are summarized in Table 2 ▸. H atoms bonded to C were placed geometrically and refined in a riding model: C—H = 0.96–0.98 Å; *U*_iso_(H) = 1.2*U*_eq_(C) or 1.5*U*_eq_(C-meth­yl). All O-bound and N-bound H atoms were initially found in difference electron-density maps, and then refined using a riding model [O—H = 0.82 Å and N—H = 0.89 Å; *U*_iso_(H) = 1.2*U*_eq_(N) and 1.5*U*_eq_(O)]. The methyl group bound to the piperazinium ring is disordered over two positions with occupancies of 0.645 (5) and 0.355 (5).

## Supplementary Material

Crystal structure: contains datablock(s) I. DOI: 10.1107/S2056989018016961/wm5472sup1.cif

Structure factors: contains datablock(s) I. DOI: 10.1107/S2056989018016961/wm5472Isup2.hkl

Supporting information file. DOI: 10.1107/S2056989018016961/wm5472Isup3.cml

CCDC reference: 1025160

Additional supporting information:  crystallographic information; 3D view; checkCIF report

## Figures and Tables

**Table 1:** Hydrogen-bond geometry (Å, °)

*D*—H⋯*A*	*D*—H	H⋯*A*	*D*⋯*A*	*D*—H⋯*A*
O5—H5⋯O7^i^	0.82	1.75	2.557 (2)	167
O2—H2⋯O1	0.82	1.78	2.535 (3)	153
N1—H1*B*⋯O7^ii^	0.89	2.48	3.046 (2)	122
N1—H1*B*⋯O6^ii^	0.89	1.91	2.790 (2)	170
N1—H1*A*⋯O3^iii^	0.89	1.94	2.811 (2)	165
C21—H21⋯O4^iv^	0.93	2.61	3.534 (3)	173
C17—H17*C*⋯F1	0.96	2.45	2.985 (3)	115
C17—H17*B*⋯F3^v^	0.96	2.53	3.380 (3)	148
C16—H16*B*⋯F1^v^	0.97	2.48	3.394 (3)	157
C16—H16*B*⋯F1	0.97	2.16	2.682 (2)	112
C16—H16*A*⋯O6^vi^	0.97	2.35	3.287 (3)	162
C14—H14⋯O6^vi^	0.93	2.59	3.448 (3)	154
C4—H4*A*⋯O4^v^	0.97	2.60	3.274 (3)	127
C2—H2*B*⋯F2	0.97	2.30	2.883 (2)	118
C2—H2*A*⋯F3	0.97	2.57	3.078 (2)	113

Symmetry codes: (i) 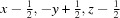; (ii) 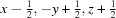; (iii) 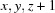; (iv) 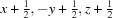; (v) 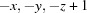; (vi) 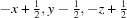.

**Table 2:** Experimental details

Crystal data	
Chemical formula	C_17_H_20_F_2_N_3_O_3_^+^·C_8_H_4_FO_4_^−^
*M*_r_	535.47
Crystal system, space group	Monoclinic, *P*2_1_/*n*
Temperature (K)	296
*a*, *b*, *c* (Å)	10.4324 (12), 16.5656 (19), 14.0448 (17)
β (°)	100.707 (3)
*V* (Å^3^)	2384.9 (5)
*Z*	4
Radiation type	Mo *K*α
μ (mm^−1^)	0.13
Crystal size (mm)	0.22 × 0.20 × 0.16

Data collection	
Diffractometer	Bruker APEXII CCD
Absorption correction	Multi-scan (*SADABS*; Bruker, 2010 ▸)
*T*_min_, *T*_max_	0.970, 0.980
No. of measured, independent and observed [*I* > 2σ(*I*)] reflections	16570, 6292, 4335
*R*_int_	0.035
(sin θ/λ)_max_ (Å^−1^)	0.711

Refinement	
*R*[*F* ^2^ > 2σ(*F* ^2^)], *wR*(*F* ^2^), *S*	0.054, 0.161, 0.98
No. of reflections	6292
No. of parameters	358
H-atom treatment	H-atom parameters constrained
Δρ_max_, Δρ_min_ (e Å^−3^)	0.29, −0.28

Computer programs: *APEX2* and *SAINT* (Bruker, 2010 ▸), *SHELXS97* and *SHELXTL* (Sheldrick, 2008 ▸), *SHELXL2014* (Sheldrick, 2015 ▸) and *publCIF* (Westrip, 2010 ▸).

## supplementary crystallographic information

### Crystal data

**Table:**

C_17_H_20_F_2_N_3_O_3_^+^·C_8_H_4_FO_4_^−^	*F*(000) = 1112
*M**_r_* = 535.47	*D*_x_ = 1.491 Mg m^−^^3^
Monoclinic, *P*2_1_/*n*	Mo *K*α radiation, λ = 0.71073 Å
*a* = 10.4324 (12) Å	Cell parameters from 5522 reflections
*b* = 16.5656 (19) Å	θ = 2.2–29.9°
*c* = 14.0448 (17) Å	µ = 0.13 mm^−^^1^
β = 100.707 (3)°	*T* = 296 K
*V* = 2384.9 (5) Å^3^	Block, colorless
*Z* = 4	0.22 × 0.20 × 0.16 mm

### Data collection

**Table:**

Bruker APEXII CCD diffractometer	4335 reflections with *I* > 2σ(*I*)
φ and ω scans	*R*_int_ = 0.035
Absorption correction: multi-scan (*SADABS*; Bruker, 2010)	θ_max_ = 30.4°, θ_min_ = 1.9°
*T*_min_ = 0.970, *T*_max_ = 0.980	*h* = −14→14
16570 measured reflections	*k* = −23→21
6292 independent reflections	*l* = −19→13

### Refinement

**Table:**

Refinement on *F*^2^	0 restraints
Least-squares matrix: full	Hydrogen site location: inferred from neighbouring sites
*R*[*F*^2^ > 2σ(*F*^2^)] = 0.054	H-atom parameters constrained
*wR*(*F*^2^) = 0.161	*w* = 1/[σ^2^(*F*_o_^2^) + (0.085*P*)^2^ + 0.686*P*] where *P* = (*F*_o_^2^ + 2*F*_c_^2^)/3
*S* = 0.98	(Δ/σ)_max_ < 0.001
6292 reflections	Δρ_max_ = 0.29 e Å^−^^3^
358 parameters	Δρ_min_ = −0.28 e Å^−^^3^

### Special details

**Table:**

Geometry. All esds (except the esd in the dihedral angle between two l.s. planes) are estimated using the full covariance matrix. The cell esds are taken into account individually in the estimation of esds in distances, angles and torsion angles; correlations between esds in cell parameters are only used when they are defined by crystal symmetry. An approximate (isotropic) treatment of cell esds is used for estimating esds involving l.s. planes.

### Fractional atomic coordinates and isotropic or equivalent isotropic displacement parameters (Å^2^)

**Table:**

	*x*	*y*	*z*	*U*_iso_*/*U*_eq_	Occ. (<1)
C2	0.2703 (2)	0.12846 (10)	0.77391 (12)	0.0390 (4)	
H2A	0.1793	0.1429	0.7531	0.047*	
H2B	0.3232	0.1710	0.7536	0.047*	
C3	0.21645 (19)	−0.01366 (11)	0.75432 (12)	0.0385 (4)	
H3A	0.2383	−0.0636	0.7249	0.046*	
H3B	0.1251	−0.0021	0.7299	0.046*	
C4	0.23908 (19)	−0.02326 (11)	0.86352 (12)	0.0391 (4)	
H4A	0.1800	−0.0637	0.8804	0.047*	
H4B	0.3277	−0.0416	0.8868	0.047*	
C1	0.30073 (19)	0.12083 (11)	0.88315 (12)	0.0398 (4)	0.645 (5)
H1	0.3925	0.1055	0.9030	0.048*	0.645 (5)
C5	0.2784 (4)	0.1988 (2)	0.9320 (2)	0.0587 (11)	0.645 (5)
H5A	0.2890	0.1903	1.0006	0.088*	0.645 (5)
H5B	0.1915	0.2177	0.9074	0.088*	0.645 (5)
H5C	0.3403	0.2383	0.9191	0.088*	0.645 (5)
C1'	0.30073 (19)	0.12083 (11)	0.88315 (12)	0.0398 (4)	0.355 (5)
H1'	0.2728	0.1712	0.9098	0.048*	0.355 (5)
C5'	0.4401 (7)	0.1112 (5)	0.9223 (4)	0.064 (2)	0.355 (5)
H5'1	0.4858	0.1593	0.9101	0.096*	0.355 (5)
H5'2	0.4734	0.0660	0.8917	0.096*	0.355 (5)
H5'3	0.4525	0.1020	0.9909	0.096*	0.355 (5)
C6	0.32692 (16)	0.05742 (10)	0.63571 (11)	0.0314 (3)	
C7	0.25702 (16)	0.02250 (11)	0.55222 (12)	0.0348 (4)	
C8	0.29167 (16)	0.02851 (11)	0.46031 (11)	0.0360 (4)	
C9	0.39760 (17)	0.07840 (11)	0.45220 (12)	0.0375 (4)	
C10	0.46920 (18)	0.11506 (11)	0.53468 (13)	0.0378 (4)	
H10	0.5396	0.1481	0.5295	0.045*	
C11	0.43600 (17)	0.10242 (11)	0.62229 (12)	0.0354 (4)	
C12	0.4354 (2)	0.09342 (13)	0.35878 (13)	0.0468 (5)	
C13	0.3573 (2)	0.05467 (14)	0.27828 (13)	0.0511 (5)	
C14	0.2580 (2)	0.00583 (15)	0.29119 (13)	0.0533 (6)	
H14	0.2103	−0.0191	0.2366	0.064*	
C15	0.3820 (3)	0.06630 (18)	0.17808 (15)	0.0690 (8)	
C16	0.12118 (19)	−0.07161 (18)	0.37829 (15)	0.0621 (7)	
H16A	0.0763	−0.0816	0.3124	0.075*	
H16B	0.0576	−0.0514	0.4148	0.075*	
C17	0.1759 (2)	−0.14920 (18)	0.4221 (2)	0.0711 (8)	
H17A	0.2399	−0.1692	0.3869	0.107*	
H17B	0.1070	−0.1880	0.4192	0.107*	
H17C	0.2161	−0.1403	0.4885	0.107*	
C18	0.15025 (16)	0.22254 (10)	0.33449 (12)	0.0331 (4)	
C19	0.25938 (16)	0.27147 (10)	0.33760 (12)	0.0331 (4)	
H19	0.2883	0.2841	0.2806	0.040*	
C20	0.32544 (16)	0.30154 (10)	0.42577 (11)	0.0325 (3)	
C21	0.28309 (17)	0.28192 (11)	0.51078 (12)	0.0352 (4)	
H21	0.3271	0.3008	0.5703	0.042*	
C22	0.17472 (17)	0.23394 (12)	0.50507 (12)	0.0389 (4)	
C23	0.10696 (17)	0.20350 (11)	0.41930 (12)	0.0374 (4)	
H23	0.0340	0.1710	0.4182	0.045*	
C24	0.07378 (18)	0.18979 (11)	0.24192 (13)	0.0398 (4)	
C25	0.44349 (18)	0.35535 (11)	0.43029 (12)	0.0390 (4)	
F1	0.14586 (10)	−0.01682 (8)	0.56051 (8)	0.0542 (3)	
F2	0.51182 (11)	0.13417 (7)	0.70199 (8)	0.0502 (3)	
F3	0.13353 (12)	0.21463 (9)	0.58853 (8)	0.0600 (4)	
N1	0.21749 (14)	0.05481 (9)	0.91112 (10)	0.0366 (3)	
H1A	0.2354	0.0485	0.9751	0.044*	
H1B	0.1339	0.0688	0.8945	0.044*	
N2	0.29693 (15)	0.05198 (8)	0.72827 (10)	0.0349 (3)	
N3	0.22256 (15)	−0.00954 (12)	0.37700 (10)	0.0466 (4)	
O1	0.52996 (17)	0.13801 (11)	0.35126 (11)	0.0634 (4)	
O2	0.4753 (2)	0.11777 (15)	0.16934 (12)	0.0864 (6)	
H2	0.5061	0.1367	0.2226	0.130*	
O3	0.3194 (2)	0.03032 (14)	0.10876 (11)	0.0906 (7)	
O4	−0.03217 (17)	0.16034 (13)	0.23770 (11)	0.0770 (6)	
O5	0.13137 (13)	0.19749 (9)	0.16727 (9)	0.0483 (3)	
H5	0.0852	0.1778	0.1192	0.073*	
O6	0.46630 (13)	0.38375 (9)	0.35255 (9)	0.0500 (4)	
O7	0.51218 (16)	0.36907 (10)	0.51142 (10)	0.0619 (4)	

### Atomic displacement parameters (Å^2^)

**Table:**

	*U*^11^	*U*^22^	*U*^33^	*U*^12^	*U*^13^	*U*^23^
C2	0.0599 (11)	0.0327 (9)	0.0257 (8)	0.0003 (8)	0.0111 (8)	−0.0032 (7)
C3	0.0511 (10)	0.0366 (9)	0.0280 (8)	−0.0052 (8)	0.0074 (7)	−0.0045 (7)
C4	0.0526 (10)	0.0355 (9)	0.0297 (8)	0.0002 (8)	0.0089 (7)	0.0023 (7)
C1	0.0533 (10)	0.0421 (10)	0.0244 (8)	−0.0045 (8)	0.0079 (7)	−0.0076 (7)
C5	0.087 (3)	0.052 (2)	0.0429 (18)	−0.0161 (17)	0.0286 (17)	−0.0244 (15)
C1'	0.0533 (10)	0.0421 (10)	0.0244 (8)	−0.0045 (8)	0.0079 (7)	−0.0076 (7)
C5'	0.066 (4)	0.091 (5)	0.033 (3)	−0.029 (4)	0.006 (3)	0.000 (3)
C6	0.0390 (8)	0.0346 (8)	0.0204 (7)	0.0060 (7)	0.0047 (6)	−0.0019 (6)
C7	0.0321 (8)	0.0470 (10)	0.0252 (8)	0.0041 (7)	0.0050 (6)	−0.0063 (7)
C8	0.0358 (8)	0.0502 (10)	0.0201 (7)	0.0152 (7)	0.0005 (6)	−0.0052 (7)
C9	0.0411 (9)	0.0482 (10)	0.0240 (8)	0.0183 (8)	0.0084 (7)	0.0030 (7)
C10	0.0411 (9)	0.0401 (9)	0.0337 (9)	0.0066 (7)	0.0111 (7)	0.0017 (7)
C11	0.0418 (9)	0.0362 (9)	0.0262 (8)	0.0040 (7)	0.0011 (7)	−0.0031 (7)
C12	0.0539 (11)	0.0578 (12)	0.0312 (9)	0.0269 (10)	0.0143 (8)	0.0094 (8)
C13	0.0613 (12)	0.0703 (14)	0.0233 (8)	0.0313 (11)	0.0119 (8)	0.0059 (8)
C14	0.0551 (12)	0.0824 (15)	0.0194 (8)	0.0299 (11)	−0.0009 (8)	−0.0088 (9)
C15	0.0890 (18)	0.0935 (19)	0.0263 (10)	0.0476 (16)	0.0155 (11)	0.0118 (11)
C16	0.0340 (10)	0.113 (2)	0.0366 (10)	0.0000 (11)	0.0000 (8)	−0.0361 (12)
C17	0.0545 (13)	0.0898 (19)	0.0727 (16)	−0.0240 (13)	0.0213 (12)	−0.0368 (15)
C18	0.0350 (8)	0.0344 (8)	0.0284 (8)	0.0011 (7)	0.0014 (7)	0.0012 (6)
C19	0.0377 (8)	0.0372 (9)	0.0236 (7)	0.0006 (7)	0.0036 (6)	0.0052 (6)
C20	0.0367 (8)	0.0331 (8)	0.0262 (8)	0.0031 (7)	0.0016 (6)	0.0046 (6)
C21	0.0406 (9)	0.0405 (9)	0.0225 (7)	0.0050 (7)	0.0010 (6)	0.0017 (6)
C22	0.0399 (9)	0.0517 (11)	0.0261 (8)	0.0057 (8)	0.0091 (7)	0.0082 (7)
C23	0.0345 (8)	0.0428 (10)	0.0344 (9)	0.0006 (7)	0.0054 (7)	0.0068 (7)
C24	0.0452 (10)	0.0417 (10)	0.0307 (8)	−0.0073 (8)	0.0023 (7)	0.0016 (7)
C25	0.0415 (9)	0.0446 (10)	0.0271 (8)	−0.0048 (8)	−0.0038 (7)	0.0082 (7)
F1	0.0424 (6)	0.0885 (9)	0.0324 (6)	−0.0148 (6)	0.0091 (5)	−0.0211 (6)
F2	0.0567 (7)	0.0588 (7)	0.0332 (6)	−0.0169 (5)	0.0034 (5)	−0.0103 (5)
F3	0.0566 (7)	0.0962 (10)	0.0305 (6)	−0.0063 (7)	0.0170 (5)	0.0108 (6)
N1	0.0432 (8)	0.0460 (8)	0.0210 (6)	0.0023 (6)	0.0072 (6)	−0.0016 (6)
N2	0.0506 (8)	0.0330 (7)	0.0219 (6)	−0.0036 (6)	0.0087 (6)	−0.0057 (5)
N3	0.0396 (8)	0.0754 (12)	0.0227 (7)	0.0143 (8)	0.0002 (6)	−0.0126 (7)
O1	0.0681 (10)	0.0823 (11)	0.0460 (8)	0.0089 (9)	0.0270 (8)	0.0126 (8)
O2	0.1021 (15)	0.1232 (18)	0.0419 (9)	0.0267 (13)	0.0340 (10)	0.0196 (10)
O3	0.1254 (16)	0.1244 (17)	0.0205 (7)	0.0381 (14)	0.0098 (9)	0.0027 (9)
O4	0.0699 (10)	0.1159 (15)	0.0427 (8)	−0.0525 (11)	0.0038 (8)	−0.0062 (9)
O5	0.0497 (8)	0.0640 (9)	0.0298 (6)	−0.0099 (6)	0.0036 (6)	−0.0103 (6)
O6	0.0497 (8)	0.0677 (9)	0.0292 (6)	−0.0176 (7)	−0.0014 (6)	0.0136 (6)
O7	0.0681 (9)	0.0778 (11)	0.0312 (7)	−0.0309 (8)	−0.0134 (7)	0.0147 (7)

### Geometric parameters (Å, º)

**Table:**

C2—N2	1.469 (2)	C12—O1	1.252 (3)
C2—C1	1.513 (2)	C12—C13	1.419 (3)
C2—C1'	1.513 (2)	C13—C14	1.353 (3)
C2—H2A	0.9700	C13—C15	1.490 (3)
C2—H2B	0.9700	C14—N3	1.349 (2)
C3—N2	1.461 (2)	C14—H14	0.9300
C3—C4	1.516 (2)	C15—O3	1.222 (4)
C3—H3A	0.9700	C15—O2	1.317 (4)
C3—H3B	0.9700	C16—N3	1.478 (3)
C4—N1	1.492 (2)	C16—C17	1.492 (4)
C4—H4A	0.9700	C16—H16A	0.9700
C4—H4B	0.9700	C16—H16B	0.9700
C1—N1	1.494 (2)	C17—H17A	0.9600
C1—C5	1.501 (3)	C17—H17B	0.9600
C1—H1	0.9800	C17—H17C	0.9600
C5—H5A	0.9600	C18—C23	1.386 (2)
C5—H5B	0.9600	C18—C19	1.392 (2)
C5—H5C	0.9600	C18—C24	1.495 (2)
C1'—C5'	1.465 (7)	C19—C20	1.393 (2)
C1'—N1	1.494 (2)	C19—H19	0.9300
C1'—H1'	0.9800	C20—C21	1.386 (2)
C5'—H5'1	0.9600	C20—C25	1.512 (2)
C5'—H5'2	0.9600	C21—C22	1.372 (3)
C5'—H5'3	0.9600	C21—H21	0.9300
C6—C7	1.387 (2)	C22—F3	1.3594 (19)
C6—N2	1.395 (2)	C22—C23	1.374 (3)
C6—C11	1.402 (2)	C23—H23	0.9300
C7—F1	1.354 (2)	C24—O4	1.200 (2)
C7—C8	1.407 (2)	C24—O5	1.308 (2)
C8—C9	1.402 (3)	C25—O7	1.249 (2)
C8—N3	1.405 (2)	C25—O6	1.252 (2)
C9—C10	1.395 (3)	N1—H1A	0.8900
C9—C12	1.460 (2)	N1—H1B	0.8900
C10—C11	1.355 (2)	O2—H2	0.8200
C10—H10	0.9300	O5—H5	0.8200
C11—F2	1.351 (2)		
			
N2—C2—C1	110.44 (14)	O1—C12—C9	121.66 (19)
N2—C2—C1'	110.44 (14)	C13—C12—C9	115.34 (19)
N2—C2—H2A	109.6	C14—C13—C12	120.31 (17)
C1—C2—H2A	109.6	C14—C13—C15	118.4 (2)
N2—C2—H2B	109.6	C12—C13—C15	121.3 (2)
C1—C2—H2B	109.6	N3—C14—C13	125.18 (19)
H2A—C2—H2B	108.1	N3—C14—H14	117.4
N2—C3—C4	110.04 (14)	C13—C14—H14	117.4
N2—C3—H3A	109.7	O3—C15—O2	122.4 (2)
C4—C3—H3A	109.7	O3—C15—C13	122.0 (3)
N2—C3—H3B	109.7	O2—C15—C13	115.6 (2)
C4—C3—H3B	109.7	N3—C16—C17	112.61 (17)
H3A—C3—H3B	108.2	N3—C16—H16A	109.1
N1—C4—C3	110.75 (14)	C17—C16—H16A	109.1
N1—C4—H4A	109.5	N3—C16—H16B	109.1
C3—C4—H4A	109.5	C17—C16—H16B	109.1
N1—C4—H4B	109.5	H16A—C16—H16B	107.8
C3—C4—H4B	109.5	C16—C17—H17A	109.5
H4A—C4—H4B	108.1	C16—C17—H17B	109.5
N1—C1—C5	111.26 (18)	H17A—C17—H17B	109.5
N1—C1—C2	107.76 (14)	C16—C17—H17C	109.5
C5—C1—C2	111.7 (2)	H17A—C17—H17C	109.5
N1—C1—H1	108.7	H17B—C17—H17C	109.5
C5—C1—H1	108.7	C23—C18—C19	120.05 (15)
C2—C1—H1	108.7	C23—C18—C24	117.27 (15)
C1—C5—H5A	109.5	C19—C18—C24	122.67 (15)
C1—C5—H5B	109.5	C18—C19—C20	120.20 (15)
H5A—C5—H5B	109.5	C18—C19—H19	119.9
C1—C5—H5C	109.5	C20—C19—H19	119.9
H5A—C5—H5C	109.5	C21—C20—C19	119.84 (16)
H5B—C5—H5C	109.5	C21—C20—C25	119.31 (15)
C5'—C1'—N1	114.0 (3)	C19—C20—C25	120.85 (14)
C5'—C1'—C2	113.4 (3)	C22—C21—C20	118.46 (16)
N1—C1'—C2	107.76 (14)	C22—C21—H21	120.8
C5'—C1'—H1'	107.1	C20—C21—H21	120.8
N1—C1'—H1'	107.1	F3—C22—C21	118.36 (16)
C2—C1'—H1'	107.1	F3—C22—C23	118.42 (16)
C1'—C5'—H5'1	109.5	C21—C22—C23	123.22 (15)
C1'—C5'—H5'2	109.5	C22—C23—C18	118.23 (16)
H5'1—C5'—H5'2	109.5	C22—C23—H23	120.9
C1'—C5'—H5'3	109.5	C18—C23—H23	120.9
H5'1—C5'—H5'3	109.5	O4—C24—O5	123.81 (17)
H5'2—C5'—H5'3	109.5	O4—C24—C18	121.90 (17)
C7—C6—N2	125.94 (15)	O5—C24—C18	114.29 (15)
C7—C6—C11	114.87 (14)	O7—C25—O6	123.89 (17)
N2—C6—C11	119.18 (14)	O7—C25—C20	118.06 (15)
F1—C7—C6	116.89 (14)	O6—C25—C20	118.06 (15)
F1—C7—C8	118.88 (15)	C4—N1—C1'	111.92 (13)
C6—C7—C8	124.17 (16)	C4—N1—C1	111.92 (13)
C9—C8—N3	119.17 (15)	C4—N1—H1A	109.2
C9—C8—C7	117.20 (15)	C1—N1—H1A	109.2
N3—C8—C7	123.56 (17)	C4—N1—H1B	109.2
C10—C9—C8	119.86 (15)	C1—N1—H1B	109.2
C10—C9—C12	118.66 (18)	H1A—N1—H1B	107.9
C8—C9—C12	121.49 (17)	C6—N2—C3	121.34 (13)
C11—C10—C9	119.92 (17)	C6—N2—C2	116.36 (13)
C11—C10—H10	120.0	C3—N2—C2	111.61 (13)
C9—C10—H10	120.0	C14—N3—C8	118.45 (18)
F2—C11—C10	118.87 (16)	C14—N3—C16	117.24 (17)
F2—C11—C6	117.42 (14)	C8—N3—C16	124.05 (16)
C10—C11—C6	123.71 (16)	C15—O2—H2	109.5
O1—C12—C13	123.00 (18)	C24—O5—H5	109.5
			
N2—C3—C4—N1	54.3 (2)	C18—C19—C20—C25	−179.48 (16)
N2—C2—C1—N1	−59.2 (2)	C19—C20—C21—C22	−1.1 (3)
N2—C2—C1—C5	178.3 (2)	C25—C20—C21—C22	179.11 (16)
N2—C2—C1'—C5'	67.9 (4)	C20—C21—C22—F3	179.86 (15)
N2—C2—C1'—N1	−59.2 (2)	C20—C21—C22—C23	0.8 (3)
N2—C6—C7—F1	3.7 (3)	F3—C22—C23—C18	−179.23 (16)
C11—C6—C7—F1	−175.55 (15)	C21—C22—C23—C18	−0.2 (3)
N2—C6—C7—C8	−179.07 (16)	C19—C18—C23—C22	−0.2 (3)
C11—C6—C7—C8	1.7 (3)	C24—C18—C23—C22	−179.07 (16)
F1—C7—C8—C9	171.85 (15)	C23—C18—C24—O4	12.6 (3)
C6—C7—C8—C9	−5.3 (3)	C19—C18—C24—O4	−166.2 (2)
F1—C7—C8—N3	−5.2 (3)	C23—C18—C24—O5	−168.50 (16)
C6—C7—C8—N3	177.68 (16)	C19—C18—C24—O5	12.6 (3)
N3—C8—C9—C10	−178.45 (15)	C21—C20—C25—O7	12.1 (3)
C7—C8—C9—C10	4.4 (2)	C19—C20—C25—O7	−167.74 (18)
N3—C8—C9—C12	1.7 (2)	C21—C20—C25—O6	−167.64 (17)
C7—C8—C9—C12	−175.47 (15)	C19—C20—C25—O6	12.5 (3)
C8—C9—C10—C11	−0.1 (3)	C3—C4—N1—C1'	−55.9 (2)
C12—C9—C10—C11	179.77 (16)	C3—C4—N1—C1	−55.9 (2)
C9—C10—C11—F2	175.67 (15)	C5'—C1'—N1—C4	−69.3 (3)
C9—C10—C11—C6	−3.9 (3)	C2—C1'—N1—C4	57.47 (19)
C7—C6—C11—F2	−176.49 (15)	C5—C1—N1—C4	−179.7 (2)
N2—C6—C11—F2	4.2 (2)	C2—C1—N1—C4	57.47 (19)
C7—C6—C11—C10	3.1 (3)	C7—C6—N2—C3	22.2 (3)
N2—C6—C11—C10	−176.24 (16)	C11—C6—N2—C3	−158.57 (16)
C10—C9—C12—O1	0.1 (3)	C7—C6—N2—C2	−119.34 (19)
C8—C9—C12—O1	179.95 (17)	C11—C6—N2—C2	59.9 (2)
C10—C9—C12—C13	−179.44 (16)	C4—C3—N2—C6	159.44 (15)
C8—C9—C12—C13	0.4 (2)	C4—C3—N2—C2	−57.40 (19)
O1—C12—C13—C14	178.65 (19)	C1—C2—N2—C6	−153.88 (15)
C9—C12—C13—C14	−1.8 (3)	C1'—C2—N2—C6	−153.88 (15)
O1—C12—C13—C15	−1.3 (3)	C1—C2—N2—C3	61.0 (2)
C9—C12—C13—C15	178.26 (17)	C1'—C2—N2—C3	61.0 (2)
C12—C13—C14—N3	1.2 (3)	C13—C14—N3—C8	1.0 (3)
C15—C13—C14—N3	−178.91 (19)	C13—C14—N3—C16	−173.3 (2)
C14—C13—C15—O3	−3.4 (3)	C9—C8—N3—C14	−2.4 (2)
C12—C13—C15—O3	176.5 (2)	C7—C8—N3—C14	174.54 (17)
C14—C13—C15—O2	176.5 (2)	C9—C8—N3—C16	171.48 (18)
C12—C13—C15—O2	−3.6 (3)	C7—C8—N3—C16	−11.6 (3)
C23—C18—C19—C20	−0.1 (3)	C17—C16—N3—C14	104.7 (2)
C24—C18—C19—C20	178.77 (16)	C17—C16—N3—C8	−69.3 (2)
C18—C19—C20—C21	0.7 (3)		

### Hydrogen-bond geometry (Å, º)

**Table:**

*D*—H···*A*	*D*—H	H···*A*	*D*···*A*	*D*—H···*A*
O5—H5···O7^i^	0.82	1.75	2.557 (2)	167
O2—H2···O1	0.82	1.78	2.535 (3)	153
N1—H1*B*···O7^ii^	0.89	2.48	3.046 (2)	122
N1—H1*B*···O6^ii^	0.89	1.91	2.790 (2)	170
N1—H1*A*···O3^iii^	0.89	1.94	2.811 (2)	165
C21—H21···O4^iv^	0.93	2.61	3.534 (3)	173
C17—H17*C*···F1	0.96	2.45	2.985 (3)	115
C17—H17*B*···F3^v^	0.96	2.53	3.380 (3)	148
C16—H16*B*···F1^v^	0.97	2.48	3.394 (3)	157
C16—H16*B*···F1	0.97	2.16	2.682 (2)	112
C16—H16*A*···O6^vi^	0.97	2.35	3.287 (3)	162
C14—H14···O6^vi^	0.93	2.59	3.448 (3)	154
C4—H4*A*···O4^v^	0.97	2.60	3.274 (3)	127
C2—H2*B*···F2	0.97	2.30	2.883 (2)	118
C2—H2*A*···F3	0.97	2.57	3.078 (2)	113

Symmetry codes: (i) *x*−1/2, −*y*+1/2, *z*−1/2; (ii) *x*−1/2, −*y*+1/2, *z*+1/2; (iii) *x*, *y*, *z*+1; (iv) *x*+1/2, −*y*+1/2, *z*+1/2; (v) −*x*, −*y*, −*z*+1; (vi) −*x*+1/2, *y*−1/2, −*z*+1/2.
